# Discovery of a very Lyman-*α*-luminous quasar at z = 6.62

**DOI:** 10.1038/srep41617

**Published:** 2017-02-02

**Authors:** Ekaterina Koptelova, Chorng-Yuan Hwang, Po-Chieh Yu, Wen-Ping Chen, Jhen-Kuei Guo

**Affiliations:** 1 National Central University, Graduate Institute of Astronomy, Taoyuan City, 32001, Taiwan

## Abstract

Distant luminous quasars provide important information on the growth of the first supermassive black holes, their host galaxies and the epoch of reionization. The identification of quasars is usually performed through detection of their Lyman-*α* line redshifted to 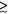
0.9 microns at z > 6.5. Here, we report the discovery of a very Lyman-*α* luminous quasar, PSO J006.1240 + 39.2219 at redshift z = 6.618, selected based on its red colour and multi-epoch detection of the Lyman-*α* emission in a single near-infrared band. The Lyman-*α* line luminosity of PSO J006.1240 + 39.2219 is unusually high and estimated to be 0.8 × 10^12^ Solar luminosities (about 3% of the total quasar luminosity). The Lyman-*α* emission of PSO J006.1240 + 39.2219 shows fast variability on timescales of days in the quasar rest frame, which has never been detected in any of the known high-redshift quasars. The high luminosity of the Lyman-*α* line, its narrow width and fast variability resemble properties of local Narrow-Line Seyfert 1 galaxies which suggests that the quasar is likely at the active phase of the black hole growth accreting close or even beyond the Eddington limit.

High-redshift quasars provide important constraints on theories of structure formation and properties of the intergalactic medium (IGM) at early cosmic epochs. The most distant of them are seen at redshifts z > 6.5 (when the Universe was less than 6% of its present age), before the end of reionization^1^. There are only seven quasars found at z > 6.5. Four were discovered in near-infrared surveys^2^,^3^. Three new quasars^4^ were discovered recently from the 3*π* Panoramic Survey Telescope and Rapid Response System^5^,^6^ (Pan-STARRS1 or PS1) sensitive up to the near-infrared y_PS1_ band (*λ*_eff_ = 0.96 *μ*m). These highest-redshift quasars host the most massive supermassive black holes (SMBHs) with masses 10^9^–10^10^ 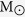 (where 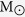 is the solar mass) accreting close to the Eddington luminosity^2^,^7^,^8^.

We used the PS1 data to search for new quasars at z > 6.5 among extremely red sources with colours z_PS1_ − y_PS1_ > 2 mag and multiple detections at several different epochs (see ‘Quasar candidate selection’ in the Methods). In our follow-up observations of these sources on November 2, 2015 we discovered a new high-redshift quasar, PSO J006.1240 + 39.2219, at z = 6.618 (see ‘Spectroscopic follow-up’ and ‘Redshift measurements’ in the Methods).

## Results

**Figure 1:**
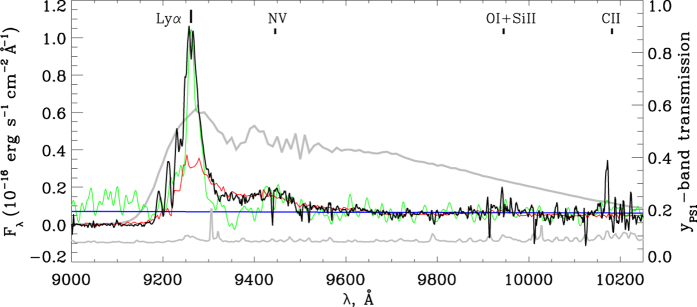
FOCAS spectrum of PSO J006.1240 + 39.2219 (black line). The displayed spectrum is smoothed with the Gaussian filter using a smoothing window of 5 Å. The sigma spectrum shown with a grey line is offset for better representation. The Ly*α* line is detected with a signal-to-noise ratio (SNR) of 34. The SNR ratios of the spectrum at the positions of the NV, OI + SiII and CII emission lines are 15, 4 and 1. The widths of the Ly*α* line, deblended and fitted with the Lorenzian profile, is estimated to be 1300 km s^−1^. The composite spectrum of z > 5.6 quasars with strong Ly*α* emission^9^ is overplotted in red. The redshifted UV spectrum of the NLS1 galaxy RE J1034 + 396 at z = 0.043 is shown in green. (The spectra are scaled to the NV emission line of PSO J006.1240 + 39.2219). The power-law continuum fit over spectral windows 9550–9900 and 10000–10150 Å (F_*λ*_ ~ *λ*^−1.1^) is shown with a blue line. The transmission curve of the PS1 y-band filter is plotted with a thick grey line.

The discovery spectrum of PSO J006.1240 + 39.2219 shows an unusually strong, compared to the continuum and, at the same time, narrow Lyman-*α* (Ly*α*) emission line with a line width of 1300 ± 90 km s^−1^ (see Fig. 1). This line width is smaller than the typical width of the emission lines produced in the Broad Line Region (BLR) of quasars. For comparison, we estimated the Ly*α* line width of the quasars known at z > 6.5 to be between 2000 and 3800 km s^−1^. The observed metal lines of PSO J006.1240 + 39.2219 (NV, OI + SiII and CII) do not exhibit the unusual strength as its Ly*α* line. As seen in Fig. 1, the peak flux ratio of the Ly*α* and NV lines of PSO J006.1240 + 39.2219 is more than twice larger than that observed in high-redshift quasars with strong Ly*α* emission^9^. The small width of the Ly*α* line of PSO J006.1240 + 39.2219 is difficult to explain as due to particularly strong absorption by neutral hydrogen (HI). The absorption features on the blue side of the line have a minor impact on its strength and shape (the core of the line is rather symmetric relative to the redshifted line wavelength). This might imply that the Ly*α* line of PSO J006.1240 + 39.2219 is dominated by a narrow-line component and its profile is intrinsically narrow. In the local Universe, narrow high-ionization, broad emission lines are also observed in Narrow-Line Seyfert 1 (NLS1) galaxies. As demonstrated in Fig. 1, the Ly*α* line of the NLS1s is much stronger than in broad-line quasars^10^. The NLS1 galaxies have smaller black holes of 10^6^–10^8^ 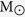 resulting in the narrow width of the BLR lines. Most of the NLS1s accrete at the super-Eddington limit^11^,^12^,^13^.

From power-law fit F_*λ*_ ~ *λ*^*α*^ to the continuum of PSO J006.1240 + 39.2219 between 9500–9900 and 10000–10150 Å, we estimated a spectral slope of *α* = −1.10 ± 0.48. By extrapolating the power law to 1450 × (1 + z) Å, we measured the absolute magnitude of the quasar at rest-frame wavelength 1450 Å to be M_1450_ = −26.1 ± 0.4, where the error includes the uncertainty in the spectral slope and redshift. Applying a bolometric correction factor of 4.4 to the ultraviolet (UV) luminosity^14^, we estimated a total quasar luminosity of 2.8 × 10^13^ 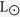 (where 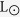 is the solar luminosity). The relation between black hole mass and bolometric luminosity for the known z ~ 6 quasars follows well the expected relation for accretion at the Eddington limit^15^. If PSO J006.1240 + 39.2219 accretes at the Eddington limit, its luminosity implies a black hole mass of 10^8^–10^9^ 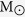
7,15. Given the relation between mass of black holes and square of the width of broad emission lines^16^, the narrow Ly*α* line of PSO J006.1240 + 39.2219 implies up to an order of a magnitude smaller black hole mass than expected from the quasar luminosity, and the super-Eddington accretion rate^7^,^15^.

**Figure 2:**
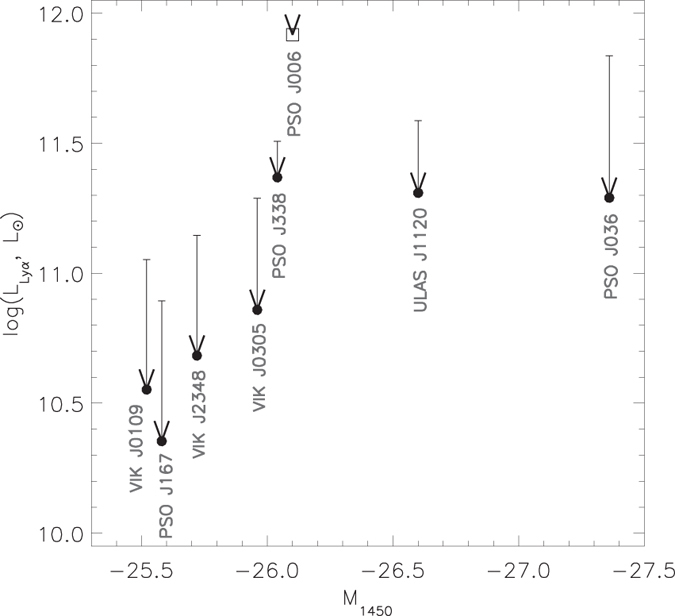
Absolute UV magnitude M_1450_ of quasars at redshidft z > 6.5 versus Lyα line luminosity. The quasars ULAS J1120 + 0641^2^, VIK J0109–3047^3^, VIK J2348–3054^3^, VIK J0305–3150^3^, PSO J167–13^4^, PSO J338 + 29^4^, PSO J036 + 03^4^ are marked with black circles, PSO J006.1240 + 39.2219 is shown with an open square. The Ly*α* line luminosity is estimated by integrating the line flux between 1204–1229 Å (~6170 km s^−1^)^41^. The upper limits correspond to the luminosity of the Ly*α* line obtained without continuum subtraction from the line flux. The continuum contribution into the Ly*α* line of PSO J006.1240 + 39.2219 is estimated to be less than 10% (within the region marked by the square) and is significantly less than in the other z > 6.5 quasars.

**Figure 3:**
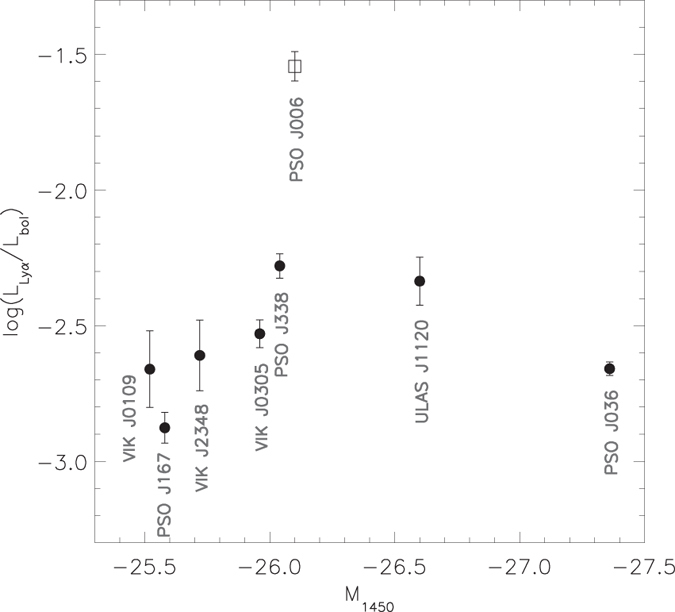
Absolute UV magnitude M_1450_ of quasars at redshidft z > 6.5 versus Lyα line luminosity expressed as a fraction of the bolometric luminosity. The object notation is similar to the previous figure.

From the spectrum of PSO J006.1240 + 39.2219 with the subtracted continuum we measured its Ly*α* line luminosity to be 0.8 × 10^12^ 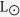, which is about 3% of the total luminosity of the quasar. We compared the luminosity of the Ly*α* line of PSO J006.1240 + 39.2219 with that of the other quasars discovered at z > 6.5. As shown in Fig. 2, the Ly*α* luminosity of PSO J006.1240 + 39.2219 is larger than the Ly*α* luminosity of the z > 6.5 quasars by more than a factor of two. It is also more than ten times larger than the Ly*α* luminosity of the most luminous Lyman Alpha Emitting galaxies (LAEs) seen during the epoch of reionization^17^. The relative contribution of the Ly*α* line into the total quasar luminosity is somewhat uncertain as due to the uncertainty in the continuum fit. However, we calculated that for power-law slopes between −0.5 and −2.5, the Ly*α* emission always dominates the UV continuum and contributes 2–4% into the bolometric luminosity of the quasar. In the other z > 6.5 quasars this contribution is only 0.1–0.5% (see Fig. 3). The rest-frame equivalent width (EW) of the Ly*α* line of PSO J006.1240 + 39.2219 is also large. Similar to the previous studies, we estimated the EW of the quasar by integrating the line flux above the continuum within 1160 < *λ*_rest_ < 1290 Å, which includes the Ly*α* and NV lines. The measured EW of PSO J006.1240 + 39.2219 is equal to 182 Å and corresponds to the high end of the equivalent width distribution of the known z > 5.6 quasars with the peak at EW ≈ 35 Å^9^. Many of the z > 5.6 quasars have absolute magnitudes brighter than that of PSO J006.1240 + 39.2219, but only small fraction of them exhibit strong Ly*α* emission lines (Their composite spectrum is shown in Fig. 1). The typical EW of these quasars is ~140 Å^9^. The EWs of the known z > 6.5 quasars are smaller than 35 Å (except for PSO J338 + 29 with 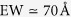
4), i.e., corresponds to the lower end of the EW distribution of the high-redshift quasars, which can be explained by stronger HI absorption at z > 6.5. The large EW of PSO J006.1240 + 39.2219 compared to these quasars implies weaker nearby intergalactic HI absorption.

**Figure 4:**
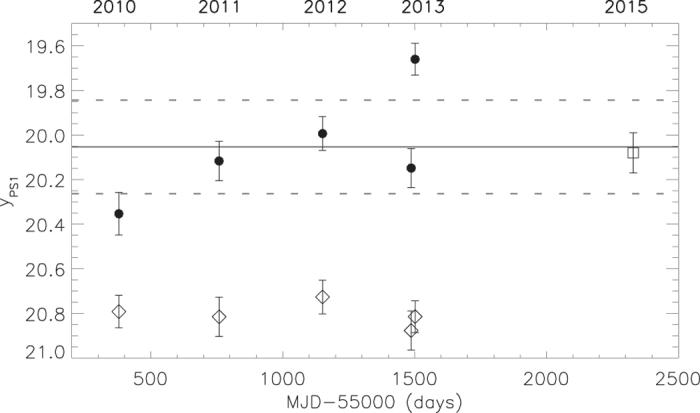
The y_PS1_-band light curve of PSO J006.1240 + 39.2219. Black points show the quasar brightness measured using aperture photometry of the PS1 images taken between June 2010 and July 2013. At each of the epochs, the quasar was observed at least two times for a total of 60s. The measured quasar flux and uncertainty at the different epochs are estimated as the mean and standard deviation of five independent quasar flux measurements relative to five nearby faint stars of ~19.5–20.0 mag. The solid and dotted grey lines correspond to a multi-epoch mean and its ±2.5*σ* error of 20.05 ± 0.21 mag. In 2010 the quasar was fainter of its mean brightness by 0.24 mag. Within 15 days in 2013, it became brighter than its mean brightness by ~0.4 mag (which is 4.6*σ* off the multi-epoch mean), showing short-term variability. The overall brightness change between 2010 and 2013 is ~0.7 mag. The open square shows the brightness of the quasar in 2015 estimated from its discovery spectrum by integrating the flux through the y_PS1_ passband. The light curve of one of the nearby faint stars of 19.65 ± 0.07 mag chosen for quasar flux calibration, is shown with diamonds. (For better representation the light curve of the star is shifted by +1.15 mag).

The multi-epoch photometry of PSO J006.1240 + 39.2219 shows the change of the quasar brightness. From the PS1 images taken between June 2010 and July 2013, we measured the y_PS1_-band brightness of the quasar at different epochs. The resulting quasar light curve is shown in Fig. 4. The brightness of the quasar at the time of our spectroscopic observations is also presented. From the quasar light curve we find that the quasar is variable on rest-frame timescales of days and months, with an amplitude exceeding its multi-epoch mean brightness by more than 2.5*σ*. The overall peak-to-peak amplitude of the observed variations is ~0.7 mag. Between 2010 and 2011, the quasar became brighter by about 0.24 mag within 50 days in the quasar rest frame. In 2013, PSO J006.1240 + 39.2219 changed its brightness from 20.15 ± 0.09 to 19.66 ± 0.07 mag over a period of ~2 days in the quasar rest frame. These high-amplitude variations are larger than brightness changes of 0.1–0.2 mag expected from the UV/optical structure function^18^ and damped random walk model^19^ of quasar variability on similar timescales. However, we note, that the Ly*α* line is ionized by the extreme UV and soft X-ray radiation which can be highly variable. For instance, the soft X-ray flux of NLS1s can change by a factor of ten on timescales of days^20^,^21^. The variation in Ly*α* emission can occur almost simultaneously with the variation of the ionizing flux on short timescales limited by hydrogen recombination time 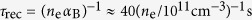 (where the typical electron density of the broad-line emitting gas is 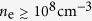
^ ^22) and the size of the Ly*α* emitting region^23^,^24^.

The y_PS1_ band measures the total flux from the Ly*α* line and nearby UV continuum. Therefore, observed variability of PSO J006.1240 + 39.2219 can be caused both by the line and continuum variations. However, the continuum brightness, corresponding to the y_PS1_-band multi-epoch mean quasar flux, is 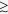
21 mag, which is below the detection limit for single exposures in the 3*π* PS1 survey 
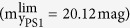
25. The relative flux contribution of the Ly*α* line into the y_PS1_-band total flux of the quasar is more than 70%. Therefore, the observed flux mostly comes from the Ly*α* line of PSO J006.1240 + 39.2219. The rapid y_PS1_-band variations of the quasar provide the evidence of variable Ly*α* emission which responds fast to the variations of the extreme UV and soft X-ray flux and, therefore, originates close to the central engine^26^. The small size of the Ly*α* emitting region, as expected from variability of the quasar, suggests a rather small mass of the central black hole^16^.

## Discussion

We reported the discovery of the Ly*α*-luminous narrow-line quasar, PSO J006.1240 + 39.2219, with the first evidence of broad-band quasar variability at high redshift. We find a similarity between the properties of PSO J006.1240 + 39.2219 and the NLS1 galaxies. The NLS1s exhibit rapid UV variability and narrow broad lines, as a result of the smaller black hole masses, an order of a magnitude smaller than the black hole masses of the broad-line quasars of the same luminosities. Similar to the NLS1s, the strong narrow Ly*α* line of PSO J006.1240 + 39.2219 without a prominent broad-line component and its short-term variability provide the evidence of the smaller black hole mass of this quasar than that expected from the luminosity - black hole mass relation.

The high luminosity of the Ly*α* line of PSO J006.1240 + 39.2219 implies that the extreme UV and soft X-ray component of the quasar continuum is strong and sustains its Ly*α* emission at a very high level^27^,^28^. We estimate the luminosity of this high-energy continuum to be L_ion_ = 1.8 L(Ly*α*)/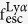 ≈ 5 × 10^12^ 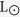
29, where we assume that the average energy of ionizing photons is 13.6 eV and escape fraction of the Ly*α* photons is 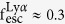. The adopted escape fraction represents the volume-averaged value that is found to evolve approximately as power law 
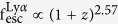
 between redshift 0 and 6^30^. We note, that the volume-averaged escape fraction includes effects of absorption by the IGM that might lead to the smaller values of 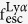 at z > 6. In spite of the uncertainty in 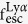, being the most luminous Ly*α* emitter, PSO J006.1240 + 39.2219 is the powerful source of ionizing radiation which likely has an important contribution into ionization of the IGM surrounding the quasar. From the observed spectrum we measure the size of the quasar ionized HII region scaled to M_1450_ = −27 to be R_NZ_ = 4 ± 1 Mpc, which is slightly larger than a near zone of 2.5–3.5 Mpc expected from the empirical relation between R_NZ_ and redshift^31^ (see ‘The near-zone size’ in the Methods).

The observed y_PS1_-band brightness variations of PSO J006.1240 + 39.2219 are likely due to variability of its Ly*α* emission as it substantially dominates the y_PS1_-band flux of the quasar. The size of the Ly*α* emitting region of PSO J006.1240 + 39.2219 inferred from the timescale of the Ly*α* rapid variations is about 2 light days. This is similar but slightly smaller than the BLR regions of the local NLS1 galaxies^24^,^32^,^33^. From the observations of reverberation time lags between the UV/X-ray continuum and Ly*α* line (and also between the UV/X-ray continuum and Balmer lines), the typical size of the Ly*α* emitting region of the NLS1s is estimated to be 3–10 light days. For comparison, the time lags (and correspondingly the BLR sizes) in broad-line quasars are 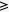
1 month^34^,^35^. We caution that if the UV continuum of PSO J006.1240 + 39.2219 is highly variable (e.g., changing by about 1 mag on short timescales) its variability imposed on the variations of the Ly*α* flux would lead to underestimation of the size of the Ly*α* emitting region inferred from the observed short-term variations.

From the similarity of PSO J006.1240 + 39.2219 with the NLS1 galaxies we infer that this quasar is young, at the early phase of its black hole and bulge formation. These Ly*α*-line luminous young quasars seen at early cosmic epochs might be capable of ionizing large volumes of gas and might play a significant role in cosmic reionization.

## Methods

### Quasar candidate selection

We searched for z_ps1_-band dropouts in the first and second internal data releases of the PS1 survey (PV1 and PV2) using the i_ps1_-, z_ps1_- and y_ps1_-band photometric catalogues. First, from the y_ps1_-band catalogue we selected point sources assuming that the difference between their point spread function (PSF) and aperture magnitudes is less than 0.3 mag, and the chi-square of the PSF fit is 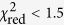. From the resulting sample we selected the z_ps1_-band dropout quasar candidates using the following criteria: z_ps1_ − y_ps1_ > 2, 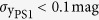 (where 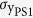 is the y_ps1_-band photometric error), i_ps1_ > 24 and z_ps1_ > 24 mag. These criteria are similar to those adopted in the previous searches of high-redshift quasars from PS1^4^. Unlike the previous works, we additionally checked for multi-epoch detections of our z_ps1_-band dropout candidates in the y_ps1_-band. The PS1 survey conducted repeated scans of the sky and provided multi-epoch photometry for detected sources in all PS1 bands. The z_ps1_-dropout candidates detected at least at two different epochs were considered by us as reliable. In this way we excluded short-lived transients and other possible artifacts from our colour-selected sample. The strongest of the multi-epoch candidates had photometric measurements at five different epochs while no detection in the z_PS1_ band. This candidate is the high-redshift quasar presented in this work. The selected high-redshift quasar candidates were also checked for the counterparts in the Wide-Field Infrared Survey Explorer all-sky source catalogue^36^ (AllWISE) within a match radius of 3 arcsec. However, none of them was detected in the WISE bands. Using this result, we place upper limits on their WISE W1 and W2 magnitudes to be W1 > 19.7 and W2 > 19.3 mag (i.e., fainter than the WISE W1 and W2 limiting magnitudes).

### Spectroscopic follow-up

We performed simultaneous photometric and spectroscopic observations of twelve z > 6.5 quasar candidates with the Subaru Faint Object Camera And Spectrograph^37^ (FOCAS) of the 8.2-m Subaru telescope. The observations were carried out on November 2, 2015. We used FOCAS long-slit mode, VPH900 grating and the SO58 order cut filter, giving us a wavelength coverage of 7500–10450 Å and a dispersion of 0.74 Å pixel^−1^. The slit was 0.8 arcsec wide resulting in a spectroscopic resolution of R ~ 1500. The seeing during the observations varied between 0.26–0.48 arcsec. Prior to spectroscopy we took acquisition images of the candidates in the FOCAS Y band. The spectra were taken only for three candidates, two of which were reliably detected during acquisition. Out of these three targets, only one had the blue-end cutoff typical for high-redshift sources. We took five 1000s through-slit exposures of this target which was identified as a quasar based on its spectrum. The stacked spectrum of the quasar and its uncertainty were calculated using median combine of the individual exposures. The quasar spectrum was absolute flux calibrated using the spectrophotometric standard star BD + 28d4211 observed on the same night.

### Redshift measurements

To estimate the redshift of the quasar, we measured the redshifted positions of the NV, OI + SiII and CII emission lines at *λ*_rest_ = 1239.85, 1305.42 and 1336.60 Å. The redshift was determined by calculating the cross-correlation function between region 9300–10200 Å of the quasar spectrum and the redshifted composite SDSS quasar spectrum^38^. The fitted wavelength range did not include the Ly*α* line. The best correlation with a correlation coefficient of 0.86 was achieved for a redshift of z = 6.618 ± 0.02.

### The near-zone size

To measure the size of the ionized HII region around the quasar, we first smoothed the quasar spectrum to a resolution of 20 Å. The transmission in the near zone was calculated by dividing the smoothed spectrum by power-law continuum F_*λ*_ ~ *λ*^−1.1^, and the Lorenzian and Gaussian fitted to the Ly*α* and NV lines. When measuring the near zone, we corrected the quasar redshift for a systematic offset of about +0.02. This systematic offset was reported between the MgII redshift and redshifts derived from high-ionization lines^39^. We measured a proper near zone of 3 ± 1 Mpc as a region where the transmitted flux drops below 10% of extrapolated continuum emission^40^. The size of the near zone scaled to M_1450_ = −27 is 4 ± 1 Mpc^31^.

## Additional Information

**How to cite this article**: Koptelova, E. *et al*. Discovery of a very Lyman-*α*-luminous quasar at z = 6.62. *Sci. Rep.* **7**, 41617; doi: 10.1038/srep41617 (2017).

**Publisher's note:** Springer Nature remains neutral with regard to jurisdictional claims in published maps and institutional affiliations.
